# Electrifying insights into cardiac arrhythmias: from molecular mechanisms to therapeutic translations

**DOI:** 10.1098/rsfs.2023.0062

**Published:** 2023-12-15

**Authors:** Mark L. Trew, Jichao Zhao

**Affiliations:** Auckland Bioengineering Institute, University of Auckland, Auckland, New Zealand

**Keywords:** cardiac arrhythmias, molecular mechanisms, therapeutic translations

## Abstract

Disruptions to normal bioelectric rate and rhythm profiles in the heart are cardiac arrhythmias. Their impacts range from minor discomforting symptoms to acute or chronic life-threatening events, with atrial fibrillation increasing the risk of stroke and heart failure, and ventricular arrhythmia associated with sudden cardiac death. To improve mechanistic understandings and advance potential approaches to treatment of arrhythmias, this Interface Focus themed issue on cardiac electrophysiology is a collection of recent studies. They investigate some of the molecular and cellular mechanisms or tissue substrates instigating and maintaining arrhythmia, and discover relevant imaging and signalling biomarkers that assess arrhythmic risks. The studies use imaging, computer simulations, machine learning and both human and animal models in their investigations exploring basic science and strategies for early recognition and improved treatment strategies.

## 1. Introduction

‘Predictions are nice, if you can make them. But the essence of science lies in explanation, laying bare the fundamental mechanisms of nature.’

M. Mitchell Waldrop, Complexity: The Emerging Science at the Edge of Order and Chaos.

There are many propositions for causes, initiators and risk stratifiers of cardiac arrhythmia. The studies presented in this special edition of Interface Focus represent a broad spectrum of approaches that add to our body of arrhythmic knowledge and understanding. They include *in vivo* [1,2], in silico [3–7], *in vitro* [8] and *ex vivo* [7,9] studies covering animal [1,7,9] and human [3,8] models and clinical imaging [2]. Most consider implications for the atria [1,2,4–6], reflecting the dominant morbidity and mortality burden of atrial fibrillation in many parts of the world, with the balance studying ventricular electrical function [3,7,9]. The motivating pro-arrhythmic disease states are similarly varied and include metabolic syndrome [1], atrial fibrillation [2,4,6,8], long-QT syndrome [3], pulmonary arterial hypertension [5] and myocardial induced heart failure [9]. While some of the studies explicitly consider a range of scales or dimensions [3,5,6], others focus on the cellular or subcellular space [7,9], the whole heart [2,4,8] or non-invasive torso-scale signals [1].

Together, the studies reported in this special edition of Interface Focus are a taster of the exciting research tracks currently pursued in the cardiac arrhythmia field. Some of these works are poised for clinical and health science translation [1,2], others present tantalizing new mechanistic insights and patient-specific pathways across complexity scales with digital models [3–7], while the final two studies seek to bridge from bench to bedside by examining the links between clinically accessible tissue structure and detailed function [8], as well as pharmaceutical interventions and outcomes [9].

## 2. New and near horizons for clinical translation and risk assessment

Heart rate variability has emerged as a measure of health and a viable biomarker for a range of cardiovascular and other diseases. It is supported through the growing and ubiquitous prevalence of wearable monitors and data-logging devices. Low heart rate variability is associated with autonomic nervous system dysregulation of cardiac activity, and this in turn is linked to patients with metabolic syndrome, a condition reaching global epidemic proportions. Long-term heart rate dynamics are significantly altered in an established animal model of metabolic syndrome and are a means for detecting early pre-clinical stages of cardiac autonomic dysfunction [1]. Metabolic syndrome significantly increases the risk of atrial fibrillation, the most common sustained arrythmia and one that is associated with substantial morbidity and mortality. While heart rhythm variation shows promise as an early indicator for metabolic syndrome, further approaches become relevant once atrial fibrillation is established. In particular, late gadolinium enhancement MRI has become the gold-standard to assess and quantify fibrosis in the left atria with atrial fibrillation driven structural remodelling. However, clinical quantification of fibrosis in the left atria has remained challenging until the development of new effective methods for processing clinical MRI [2]. Together, the combination of early metabolic syndrome detection through accessible heart rate variability biomarkers [1] and later post atrial fibrillation assessment of structural remodelling progression and arrhythmic risk [2] are both promising for clinical translation in the near term.

## 3. Bridging scales and probing mechanisms with new tools

Arrhythmias manifest as re-entrant electrical waves at a scale commensurate with cardiac chamber dimensions and underlying tissue properties. However, the origins of arrhythmia can be traced in some cases to alterations many scales below this, at the level of genetic mutations. With computer modelling, a specific mutation affecting electrical repolarization via the hERG potassium channel on cell membranes is found to enhance ventricular scale repolarization dispersion, at the same time increasing vulnerability to conduction block and causing a high density of wavelets from destabilized rotor waves [3]. These are pro-arrhythmic features and contributors to sustained arrhythmia. Having identified a genetic mutation of interest, being able to encode its likely effects on cell electrophysiology into a computer model of whole ventricle biophysics and then use this to understand pro-arrhythmic risk is a powerful tool. These approaches also have implications for drug discovery and show how key genetic or drug-induced alterations at the cellular level might be incorporated into personalized heart models. Personalized heart models can be used to guide individual anti-arrhythmic therapies, predict patient outcomes or to construct populations for in-silico trials. However, the process of obtaining and digitizing personalized anatomy introduces a raft of new challenges. A new open-source tool for building personalized atrial models at scale from common clinical imaging modalities shows how this is possible and reliable for capturing atrial fibrillation dynamics [4]. The goal is an ecosystem for digital twins in healthcare [4].

Arrhythmias in the right side of the heart often occur downstream to common respiratory diseases. Despite knowing this, the mechanisms of these arrhythmia have been understudied. Atrial fibrillation is a frequent occurrence in patients with pulmonary arterial hypertension, where the right atria is dilated and remodelled due to increased pressure load. Across the atrial chambers, heterogeneous gene expressions of calcium handling, sodium and potassium channels and connections have been found. Incorporating remodelled left and right atria up- and downregulated genes into computer models shows that the right atria is preferentially susceptible to afterdepolarizations, slowed conduction and shortened action potential durations [5]. Coupled with increased fibrosis and autonomic system disorders, the atria are particularly vulnerable to arrhythmia in pulmonary arterial hypertension [5].

There are outstanding questions concerning the triggers for arrhythmia. Spontaneous calcium release events have been proposed as a mechanism. In single cells, the calcium transient can activate the sodium-calcium exchanger, causing delays after depolarizations and potentially a triggered action potential. In well-coupled tissue there is a low probability that stochastic calcium release events will be sufficiently concurrent to overcome the electrotonic load of neighbouring cells. However, comprehensive computer models show that patchy fibrosis, consistent with atrial fibrillation, is a trigger substrate as focal excitations from calcium events are more likely due to reduced electronic load with altered cellular coupling [6]. This is exacerbated by myocyte-fibroblast coupling driving unidirectional conduction and initiating spontaneous arrhythmia [6].

For these insights into the pro-arrhythmic potential of spontaneous calcium release in a fibrotic atrial substrate, a reduced model of stochastic calcium events is necessary for tractable computer simulations [6]. It is challenging to strike a balance between ionic current model fidelity and the computational feasibility of phenomenological models in computer simulations of electrical arrhythmia over multiple seconds of model time. A promising approach is to capture the key dynamics of electrical activation using a physics-based mathematical model and augment this throughout the action potential with a trained deep-learning data model [7]. The training data includes both high-fidelity biophysical models and optical action potentials, and the resulting hybrid models robustly capture and predict electrical activity, promising fast parameterization and predicted activation sequences [7]. Such models could ideally couple with personalized digital twin atrial models [4].

## 4. Tilting the bench towards the bedside

While many studies are by necessity designed as bench studies, they are tilted towards the clinical bedside by teasing out mechanisms downstream to either imaging biomarkers or pharmaceutical treatments. Explanted donor hearts with a history of atrial fibrillation show that for induced atrial fibrillation, localization of arrhythmia drivers in the right atria are associated with variable wall thickness in that chamber [8]. Wall thickness measures capture the architecture of bundles and clusters of many cells and thus are amenable to research [8] and non-invasive clinical imaging [2,6]. At the subcellular scale, altered architecture of t-tubule structures has been found in heart failure. An emergent hypothesis is that fibrosis drives t-tubule remodelling and, hence, aberrant calcium handling with subsequent pro-arrhythmic impacts. Clinically, the anti-fibrotic drug pirfenidone, used to treat pulmonary fibrosis, has demonstrated effectiveness in heart failure patients. However, there is limited evidence showing how this treatment option affects t-tubule remodelling. In a myocardial infarct induced heart failure model, super-resolution imaging shows relatively short-term use of pirfenidone and increases the area and length of t-tubules near the infarct significantly compared to controls [9].

## 5. Summary

While much has been discovered about cardiac arrhythmia over many years of careful study, it is clear there remain fertile paths toward exposing more of the fundamental mechanisms of arrhythmogenesis and sustained arrhythmia. A selection of these paths has been reported in this special edition. They demonstrate exciting and ongoing exploration into both fundamental and ensemble mechanisms underlying arrhythmogenesis in all chambers of the heart. However, the challenge is not complete, and the findings of these studies set the scene for many new and intriguing follow-up research questions.

## Data Availability

This article has no additional data.
